# Oral Health Status in Marfan Syndrome: A Systematic Review and Meta-Analysis of 353 Cases

**DOI:** 10.3390/ijerph19095048

**Published:** 2022-04-21

**Authors:** Mohammad Khursheed Alam, Ahmed Ali Alfawzan, Deepti Shrivastava, Kumar Chandan Srivastava, Haytham Jamil Alswairki, Samir Mussallam, Huda Abutayyem, Naseer Ahmed

**Affiliations:** 1 Orthodontics, Preventive Dentistry Department, College of Dentistry, Jouf University, Sakaka 72345, Saudi Arabia; 2 Department of Preventive Dentistry, College of Dentistry in Ar Rass, Qassim University, Ar Rass 52571, Saudi Arabia; ah.alfawzan@qu.edu.sa; 3 Periodontics, Preventive Dentistry Department, College of Dentistry, Jouf University, Sakaka 72345, Saudi Arabia; sdeepti20@gmail.com; 4 Department of Oral & Maxillofacial Surgery & Diagnostic Sciences, College of Dentistry, Jouf University, Sakaka 72345, Saudi Arabia; drkcs.omr@gmail.com; 5 School of Dental Sciences, Universiti Sains Malaysia, Kota Bharu 16150, Malaysia; hitham.swerki@gmail.com; 6 Orthodontist, Private Clinic in Dubai, Dubai P.O. Box 65882, United Arab Emirates; sameer444@hotmail.com; 7 Department of Clinical Sciences, Center of Medical and Bio-Allied Health Sciences Research, College of Dentistry, Ajman University, Ajman P.O. Box 346, United Arab Emirates; h.abutayyem@ajman.ac.ae; 8 Department of Prosthodontics, Altamash Institute of Dental Medicine, Karachi 75500, Pakistan; naseerahmed@student.usm.my; 9 Prosthodontics Unit, School of Dental Sciences, Health Campus, Universiti Sains Malaysia, Kota Bharu 16150, Malaysia

**Keywords:** Marfan syndrome, orofacial health status, oral health, systematic review, meta-analysis

## Abstract

This meta-analysis aimed to compare Marfan syndrome (MFS) patients with non-MFS populations based on orofacial health status to combine publicly available scientific information while also improving the validity of primary study findings. A comprehensive search was performed in the following databases: PubMed, Google Scholar, Scopus, Medline, and Web of Science, for articles published between 1 January 2000 and 17 February 2022. PRISMA guidelines were followed to carry out this systematic review. We used the PECO system to classify people with MFS based on whether or not they had distinctive oral health characteristics compared to the non-MFS population. The following are some examples of how PECO is used: P denotes someone who has MFS; E stands for a medical or genetic assessment of MFS; C stands for people who do not have MFS; and O stands for the orofacial characteristics of MFS. Using the Newcastle–Ottawa Quality Assessment Scale, independent reviewers assessed the articles’ methodological quality and extracted data. Four case-control studies were analyzed for meta-analysis. Due to the wide range of variability, we were only able to include data from at least three previous studies. There was a statistically significant difference in bleeding on probing and pocket depth between MFS and non-MFS subjects. MFS patients are more prone to periodontal tissue inflammation due to the activity of FBN1 and MMPs. Early orthodontic treatment is beneficial for the correction of a narrow upper jaw and a high palate, as well as a skeletal class II with retrognathism of the lower jaw and crowding of teeth.

## 1. Introduction

**Figure 1:**
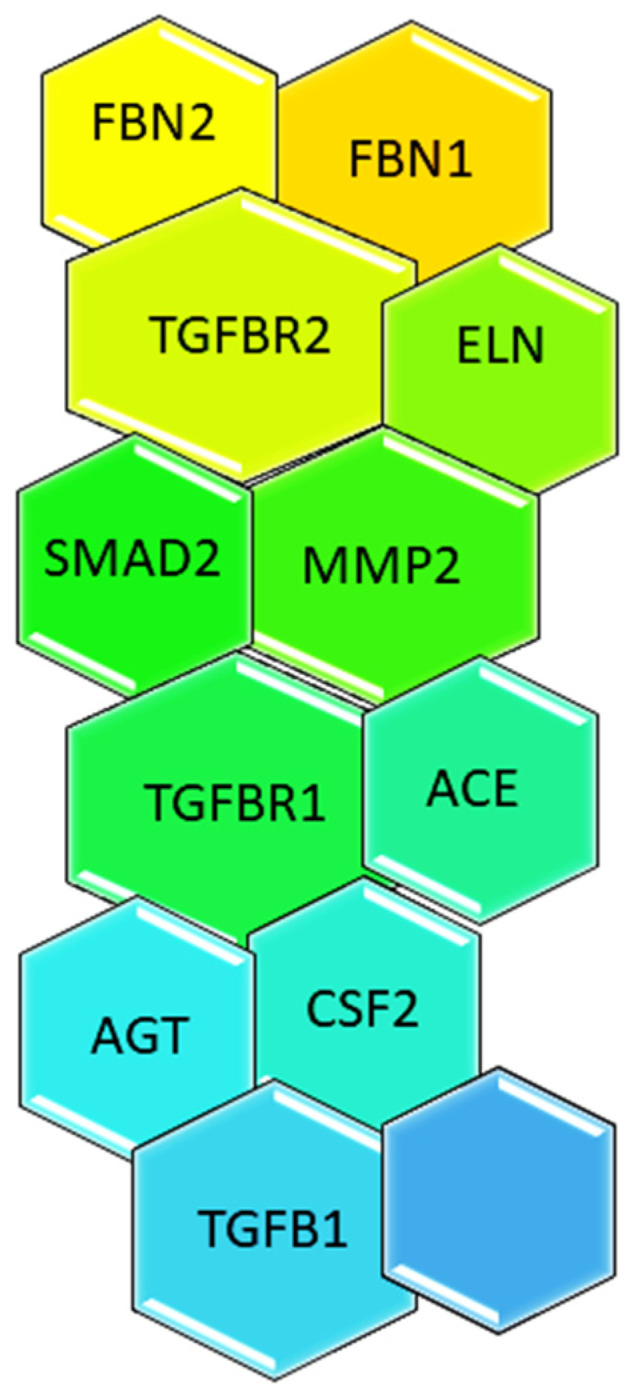
Genes and mutations commonly found in MFS.

Marfan syndrome (MFS) is an autosomal dominant genetic condition [1]. It was first described by the French pediatrician Antonin Marfan in 1896 [2]. MFS patients have problems in a variety of organs, but the problems of the cardiovascular system are the most devastating [3,4]. Dissecting thoracic aortic aneurysm (TAA) is the most life-threatening clinical symptom in adults. Infants with severe mitral valve prolapse, valvular regurgitation, and aortic root dilatation in the presence of congestive heart failure are less frequently detected [5]. The main cause is autosomal recessive condition 15q21.1, which occurs when the fibrillin-1 (FBN1) gene on chromosome 15 is damaged. FBN1 is a big part of the extracellular matrix’s microfibrils, and this gene makes them [6]. FBN1 is also made by this gene. It is thought that the FBN1 monomers make the microfibrils, which then make the elastin fibers, which connect, attach, and protect tissues and organs [3]. Studies have demonstrated that FBN1 is able to produce TFG, an important inflammatory mediator, and fibrosis, along with MMP-2 and MMP-9 activation [7]. Patients with MFS have ELN, FBN1, and TGFBR2 mutations, as well as EGF, AGT, and TGFB1 gene mutations, which are the most common in MFS patients (Figure 1) [6,7].

**Figure 2:**
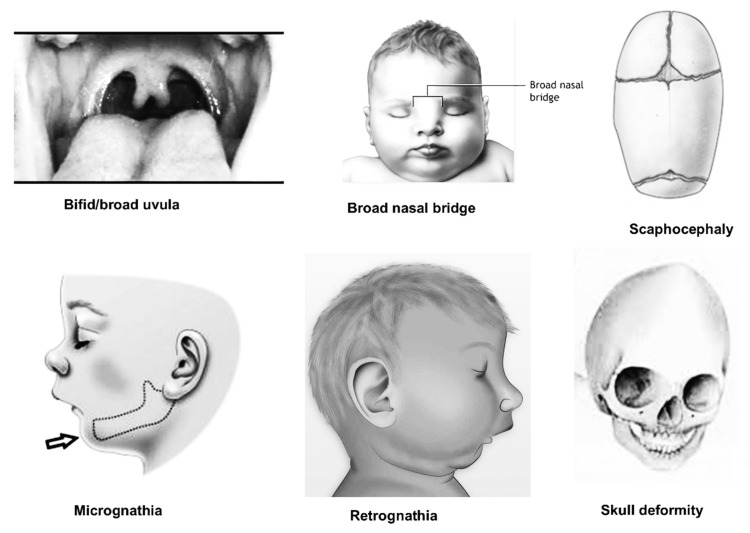
Common oral features found in MFS (Courtesy of Dr. M. K. Alam).

Oral signs of MFS include retrognathia, dolichocephaly, a high palatal vault, crowded teeth, TMJ problems, and partial anodontia, in addition to the previously stated multisystemic characteristics (Figure 2) [8,9]. Periodontal ligament dysfunction has been linked to MFS, suggesting that FBN-1 microfibril production plays a key role in periodontal ligament formation. Furthermore, oxytalan fibers, the elastic fibers of the periodontal ligament known as FBN-1 microfibrils, do not contain substantial levels of elastin [10]. If the tooth surface is covered with a biofilm that alters the periodontal ligament and/or the extracellular matrix, this can lead to significant and unfavorable effects on periodontal tissues, such as increased vulnerability and an inflammatory response, which ultimately result in tissue disintegration [11].

People with MFS’s oral health are still a subject of much debate. These patients, who are being treated by dentists, are at risk for cardiovascular complications as well, so they need to be informed about their oral health. In order to avoid bacteremia, we aim to find more frequent oral health problems that need to be treated with priority.

## 2. Materials and Methods

### 2.1. Search Strategy

**Figure 3:**
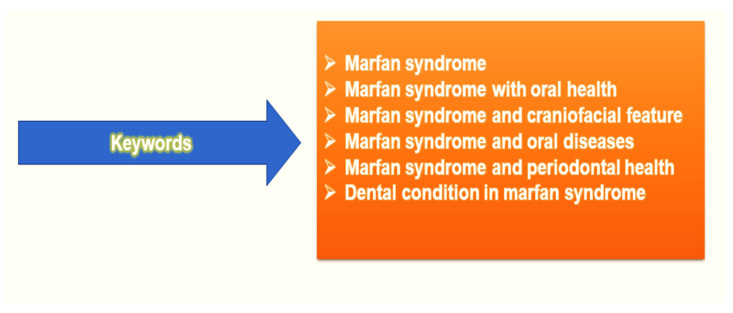
Keywords used for data searching.

**Figure 4:**
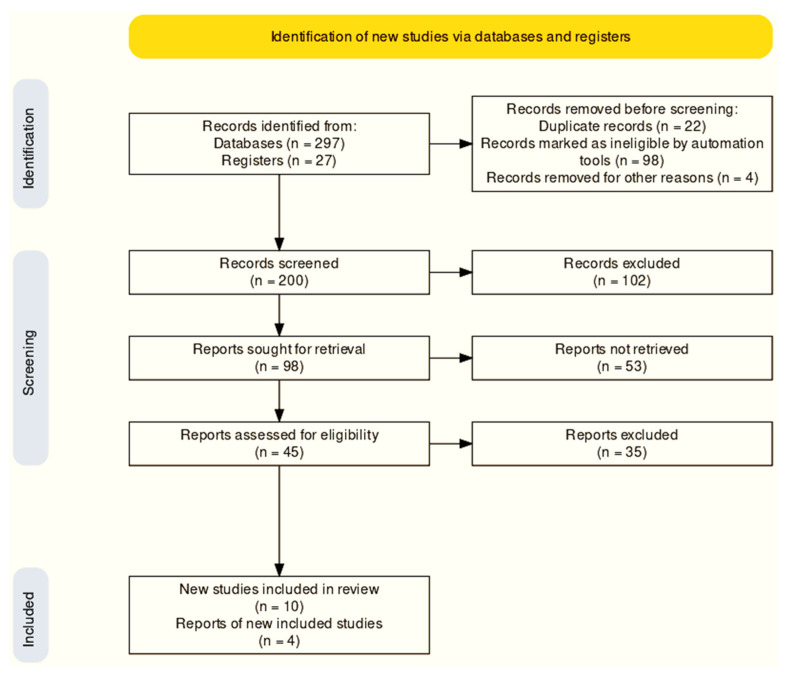
PRISMA flowchart.

Articles published between 1 January 2000 and 17 February 2022 were searched for in PubMed, Google Scholar, Scopus, Medline, and Web of Science. This study made use of keyword and Boolean operator “AND” combinations (Figure 3). MFS with oral health and English-language publications published in peer-reviewed journals were included in the search, which included full-text articles. Clinical case reports, pilot experiments, and bibliographic reviews were omitted from the list of studies that were deemed insufficient. The many steps (identification, screening, and included studies) required in making the final selections may be seen in Figure 4. In accordance with the PRISMA standards, this study was registered in the PROSPERO database (registration number: CRD42021282283) [12].

### 2.2. Study Selection Criteria

PECO [13] was utilized to classify MFS patients based on their orofacial features in comparison to non-MFS patients. PECO is used in the following ways: P denotes someone who has MFS; E stands for a medical or genetic assessment of MFS; C stands for people who do not have MFS; and O stands for the orofacial characteristics of MFS. Study participants with and without MFS satisfied inclusion criteria in case-control, cross-sectional, and cohort studies that examined DMFT, bleeding on probing, gingival index, and periodontal pocket depth. It was decided by consensus that any disputes in the results would be handled by two independent researchers (M.K.A and K.C.S). When the first two assessors could not come to an agreement (D.S.), a third was called in. Searches of the articles’ bibliographic references were also handled manually.

### 2.3. Data Extraction and Quality Assessment

Two researchers (M.K.A. and K.C.S.) went through each publication and gathered the following data: authors, year, country, number of participants, gender, and any concluding notes. DMFT, bleeding on probing (BOP), pocket depth (PD), and a variety of other parameters were excluded from the meta-analysis due to their lack of consistency across at least three studies. The mean and standard deviation were reported for each measurement. It was determined that the articles’ methodological quality was assessed by three examiners, two working together (M.K.A. and K.C.S.) and one working alone (D.S.), with the help of the Newcastle–Ottawa Quality Assessment Scale (NOS) [14]. Visual risk of bias was assessed using the ROBIN-I scale and funnel plots [15].

### 2.4. Statistical Analysis

There was a separate meta-analysis for each of the protentional findings. Because individual publications’ cephalometric measurements vary, a meta-analysis could only be performed if a mean datum was supplied in at least three articles. A random-effects model was adopted because of the evidence of heterogeneity in the individual studies. A pooled effect size (mean difference) and a 95 percent confidence interval were assigned to each outcome. The Q statistic and I^2^ index were used to look at the effect size heterogeneity [16]. Results of the Q statistic (*p* > 0.05) indicated that the population was not homogeneous. Results showed that indices of heterogeneity I^2^ ranging from 25% to 75% indicated low to moderate to significant heterogeneity, respectively. R studio and MedCalc (version 19.3) were used for all statistical studies (metafor package).

## 3. Results

### 3.1. Selection of Studies

There were 324 papers (databases: 297; registers: 27) retrieved from databases such as PubMed, Web of Science, Scopus, Medline, and Google Scholar. The remaining 200 papers were checked again after 124 were eliminated in the detection phase (reviews, summary documents, non-human, editorials, case reports, commentaries, letters, and duplicate studies). A total of 200 records were screened for further evaluation, where 102 records were excluded. Ninety-eight records were primarily sought for retrieval. Fifty-three studies were not able to be retrieved. A total of 35 out of 45 studies were deemed unsuitable because of unacceptable data formats. Figure 4 shows the 10 studies that were included in this analysis based on the research objectives and inclusion and exclusion criteria, and the entire text of all included articles was obtained. In the meta-analysis synthesis, only five studies were incorporated.

### 3.2. Study Characteristics

Table 1 [8,9,17,18,19,20,21,22,23,24] lists the most important aspects of the studies that were considered. Peer-reviewed journals were used to publish all of the investigations. A total of 3 of the 10 studies were carried out in Germany [8,17,20] and Italy [18,19,21], 2 in Japan [22,23], and 2 in Belgium [9,24]. The measurement of gingival and periodontal clinical parameters was the most employed method in the studies. In total, 353 cases and 929 controls were included in all studies. While Staufenbiel [8] reported the most cases, Venza [19] reported the fewest. Plaque and caries are more common in the mouths of people with MFS, and this leads to an overall inflammation of the oral cavity. The palatal length and height were both significantly increased in MFS, as was the height of the maxilla-alveolar processes. Comparing MFS patients to non-MFS patients, the cranial base, maxillary complex, mandibular body, and jaws’ connection to each other were found to be significantly different.

### 3.3. Meta-Analysis

**Figure 5:**
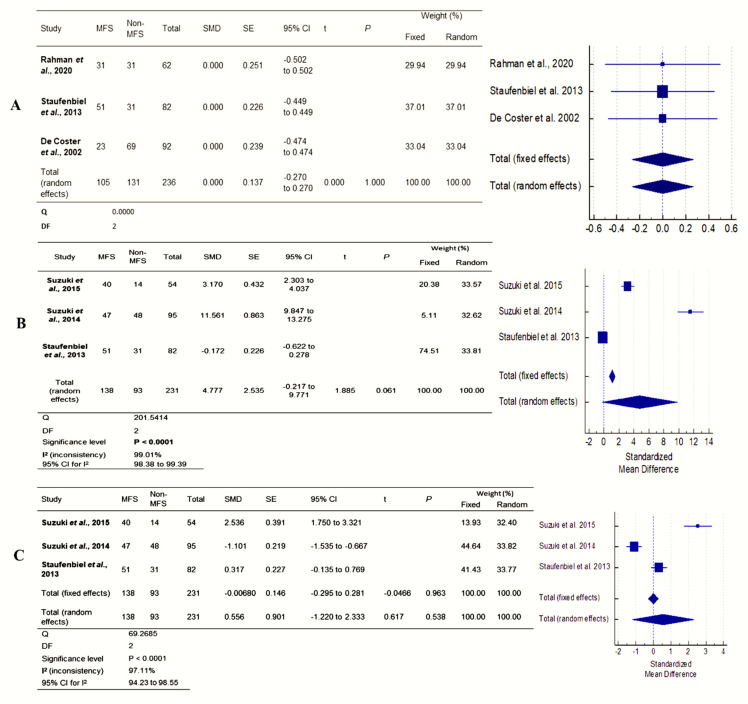
Forest plot of (**A**) DMFT, (**B**) pocket depth (PD), and (**C**) bleeding on probing (BOP) [8,9,17,22,23].

Figure 2 shows the results of four studies. Both MFS and non-MFS patients had different BOP and periodontal PD. Because there was relatively little research, interpreting Q data required caution. The I^2^ index measures effect size heterogeneity more precisely. The forest plots were created to show the studies’ heterogeneity. Only BOP and periodontal PD outcomes were heterogeneous. Each outcome was subjected to subgroup analyses to determine differences in effect sizes. The effect size and heterogeneity of the DMFT (95% CI: −0.27 to 0.27; *p* = 1.000), BOP (MD = 4.77; 95% CI: −0.27 to 9.77; I2 = 99.01%; *p* = 0.001), and periodontal pocket depth (MD = 0.55; 95% CI: −1.22 to 2.33; I2 = 97.11%; *p* = 0.001) were all significant except for DMFT (Figure 5). Compared to non-MFS patients, MFS patients had moderate to severe gingivitis and periodontitis.

### 3.4. Risk of Bias

**Figure 6:**
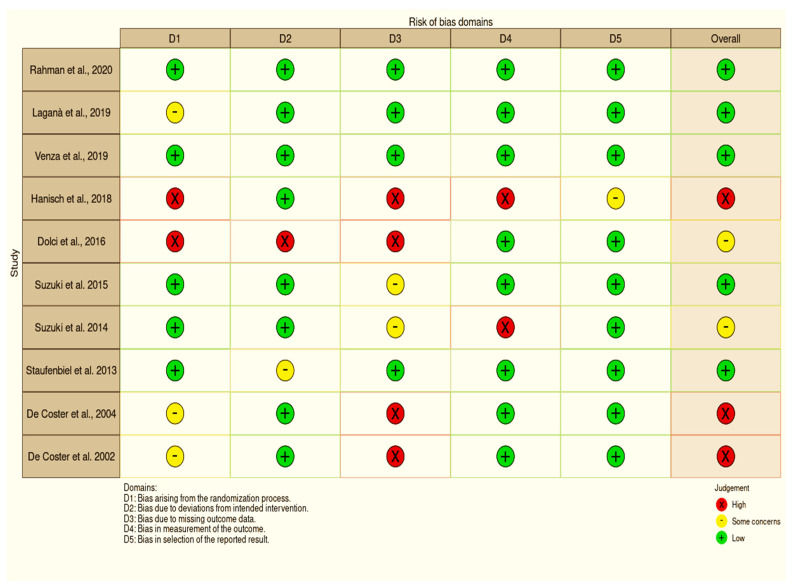
Risk of bias assessment visualization [8,9,17,18,19,20,21,22,23,24].

**Figure 7:**
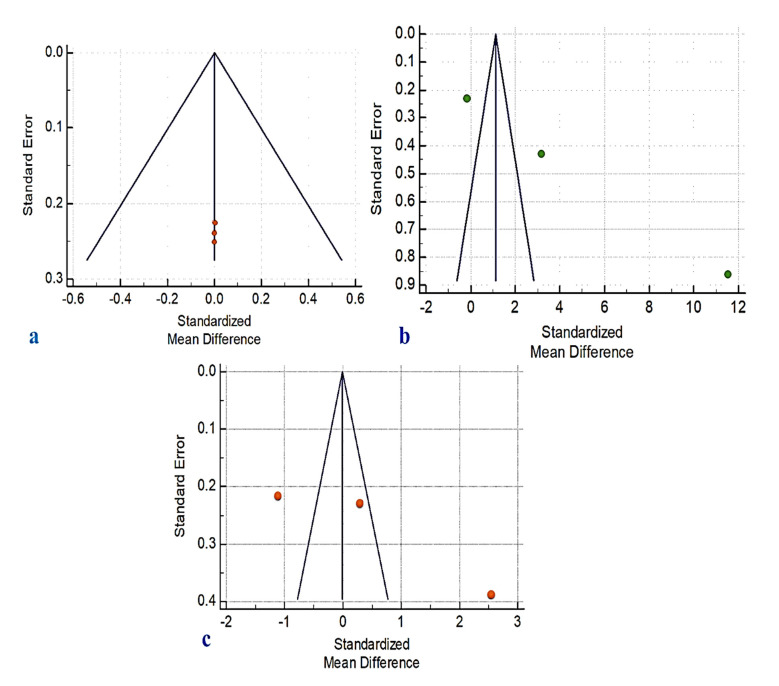
Funnel plot for publication bias in (**a**) DMFT, (**b**) pocket depth, and (**c**) bleeding on probing.

A NOS analysis was performed to determine the degree of bias present in the studies that were considered. A perfect score was achieved by four of the articles that were evaluated (Table 2). Using ROBINS-I risk of bias tools (Figure 6) and a funnel plot (Figure 7), we were able to identify publication bias in the research. This shows the link between the included studies’ effect estimates and their precision, or study size. If the funnel plot has asymmetrical lines, there is a lack of homogeneity and reporting bias. Poor methodological design and small sample sizes can also lead to asymmetry. Language bias (English-only) and citation bias may also be factors in this imbalance.

## 4. Discussion

In this systematic review and meta-analysis study, the orofacial status of MFS patients was compared to that of non-MFS patients in the general population. During the chosen time period, all English-language papers were included in the literature search to ensure that no relevant information was overlooked. At least three independent studies were required for a meta-analysis to include their findings. Meta-analysis results should be interpreted with caution. There was a significant difference in BOP rates, mean PD, and periodontal status between MFS groups and the general population (Figure 6). However, due to the possibility of bias in each of the four studies, these findings should be interpreted with care. To avoid bias, longitudinal designs only allow for actual cause-and-effect relationships, which may have weakened their internal validity. This problem could have an impact on the studies’ dependability and quality. As a result, no sample size calculations were published in any of the studies considered. A type II error (failure to reject a false null hypothesis) may have happened due to insufficient statistical power in all of these investigations, given the limited number of participants. Another possible problem with this work is that a study of publication bias (such as a funnel plot) is not properly shown because there are not many publications to look at.

Rahman et al. studied 31 children with MFS to determine the prevalence of dental caries. Patients with MFS did not have a higher rate of dental caries than healthy youngsters [17]. According to De Coster, MFS patients had a worse gingival index than control subjects [9]. According to the BOP and periodontal pocket depth in this meta-analysis, there was a significant difference between the two groups when it was used to measure periodontal inflammation. This could be due to the presence of other variables that could be confounding. Patients with MFS are more likely to have crowded teeth than those without the condition [8,25]. In these instances, it is undeniable that adequate oral hygiene might be difficult to maintain. This suggests that patients with MFS may have higher levels of inflammation due to their malocclusion and the condition itself [26].

According to Venza and colleagues, individuals with MFS have a higher prevalence of plaque and more extensive inflammation of the oral cavity [19]. Furthermore, there were no symptoms of severe periodontal attachment loss on many teeth in participants with MFS. Researcher Staufenbiel used a comprehensive periodontal status, which included probing pocket depth, clinical attachment level, and bleeding on probing, to investigate MFS patients. The patients with MFS did not have a higher risk of periodontal disease based on the mean values of the clinical attachment level [8]. According to Suzuki et al., a study published in 2015, MFS patients were more likely to suffer from periodontitis than those in the control group at the same age. The patients with MFS had more severe periodontitis than those in the control group [22]. The pathogenesis of cardiovascular problems in MFS patients may be influenced by periodontitis, as well [23].

Patients with MFS often have a narrow upper jaw and a high palate, as well as a skeletal class II with retrognathism of the lower jaw and crowding of teeth [24]. The prevalence of pulp stones and pulp calcifications was also found to be higher in patients with MFS [27,28]. Although these changes are not considered to be a disease, root canal treatments may be more prone to complications. Based on clinical and radiological findings, patients with MFS had a higher prevalence of craniomandibular dysfunction [29,30]. Periodontitis is caused by microorganisms in the subgingival biofilm and lifestyle [31,32]. Periodontitis is a chronic condition with many facets, which may explain the disparity [22]. A person’s lifestyle may be more important than their medical condition. However, it is plausible that MFS patients are more prone to periodontal tissue inflammation due to the activity of FBN1 and MMPs [33]. Patients should closely follow a maintenance therapy program to avoid or minimize periodontal disease.

This study’s goal was to compile all of the existing research on a particular issue and organize it into manageable categories [34]. This was conducted to characterize the available data and recommend future studies that are as objective as possible [35]. Research into the role of MFS as an independent risk factor in the development, progression, and severity of oral diseases in healthy people will be necessary to validate or reject our findings. This means that future studies should use a long-term design and pay attention to both the group and individual characteristics of the population being studied.

## 5. Conclusions

Our meta-analysis links MFS to gingivitis and periodontitis. Because only four analytical case-control studies were included, longer research is required to establish a causal association. A well-planned dental monitoring and early necessary orthodontic treatment plan are also required for better outcomes.

## Figures and Tables

**Table 1:** Characteristics of the studies included in the systematic review.

No	Author (Year)	Country	Study Design	Participants	Age Range (Years)	Sex (M/F)	Method	Findings
1	Rahman et al., 2020 [17]	Germany	Case-control	MFS: 31 Non-MFS: 31	MFS: 8.77 ± 3.72 Non-MFS: 9.77 ± 3.72	MFS = M:13; F:18 Non-MFS = M:13; F:18	DMFT Caries restoration index Hygiene index	• Children and adolescents with MFS did not show a higher caries experience compared to a systemically healthy control group.
2	Laganà et al., 2019 [18]	Italy	Case-control	MFS: 28 Non-MFS: 23	MFS: 8.4 ± 2.3 Non-MFS: 8.9 ± 2.9	MFS = M:17; F:11 Non-MFS = M:12; F:11	Zymography Western immunoblot	• Indicators of MMP activity included saliva and gingival crevicular fluid (GCF). • Periodontal matrix and inflammatory response can be significantly altered by even small variations in MMP-13 activity.
3	Venza et al., 2019 [19]	Italy	Case-control	MFS: 16 Non-MFS: 20	MFS: 9.4 ± 2.3 Non-MFS: 10.0 ± 2.6	MFS = M:9; F:7 Non-MFS = M:8; F:12	Plaque index Bleeding on probing (BOP) Modified periodontal screening and recording	• Patients with MFS revealed a higher presence of plaque and consequently a generalized inflammation in the oral cavity.
4	Hanisch et al., 2018 [20]	Germany	Cross-sectional survey	MFS: 51	MFS: 42.73 ± 14.50	MFS = M:17; F:11	OHIP-14 (Oral Health Impact Profile) questionnaire	• People with Marfan syndrome had a higher OHIP score than the German general public, and the vast majority of responders reported oral symptoms as a result of the disorder. Female individuals had lower OHIP-14 scores than male participants.
5	Dolci et al., 2016 [21]	Italy	Case-control	MFS: 49 Non-MFS: 661	MFS: 18–60 Non-MFS: matched	MFS = M:18; F:31 Non-MFS = M:332; F:329	50 soft-tissue facial anthropometric landmarks Three-dimensional facial image using a stereophotogrammetric system	• The mandibular ramus was shorter in 96% of MFS participants compared to non-MFS subjects, and facial divergence was larger in 100% of MFS subjects.
6	Suzuki et al., 2015 [22]	Japan	Case-control	MFS: 40 Non-MFS: 14	MFS: 34.9 ± 2.0 Non-MFS: 32.4 ± 2.2	MFS = M:23; F:17 Non-MFS = M:10; F:4	Periodontal status, BOP, Pocket depth	• The MFS patients and the control group had comparable pocket depths and bleeding on probing. MFS patients had a high rate of periodontitis and cardiovascular problems.
7	Suzuki et al., 2014 [23]	Japan	Case-control	MFS: 47 Non-MFS: 48	MFS: 35.2 ± 1.8 Non-MFS: 33.5 ± 0.9	MFS = M:29; F:18 Non-MFS = M:29; F:19	Periodontal status, BOP, Pocket depth	• Periodontitis influenced the pathophysiology of cardiovascular complications in MFS patients. A specific periodontal pathogen might be a crucial therapeutic target to prevent CVD development.
8	Staufenbiel et al., 2013 [8]	Germany	Case-control	MFS: 51 Non-MFS: 31	MFS: 40.20 ± 15.32 Non-MFS: 40.29 ± 13.94	MFS = M:21; F:30 Non-MFS = M:14; F:17	DMFT Periodontal status, BOP, Pocket depth	• Due to their overcrowded teeth, MFS patients had a tendency to display greater indicators of inflammation. For this reason, a six-month interval between professional dental cleanings is recommended to minimize the bacterial biofilm in the oral cavity, which in turn reduces the risk of systemic disorders, such as endocarditis.
9	De Coster et al., 2004 [24]	Belgium	Case-control	MFS: 17 Non-MFS: 32	MFS: 31.4 ± 11.4 Non-MFS: matched	MFS = M:23%; F:77% Non-MFS = M:23%; F:77%	Lateral cephalometric radiographs Fourteen landmarks	• The cranial basis, the maxillary complex, the mandible body, and the jaws’ relationship to the cranial base and to each other showed significant disparities in the control group. • In MFS, the palatal height and palatal length were considerably bigger, and the height of the maxilla-alveolar processes was significantly associated to both.
10	De Coster et al., 2002 [9]	Belgium	Case-control	MFS: 23 Non-MFS: 69	MFS: 9–53 Non-MFS: 9–53	MFS = M:14; F:9 Non-MFS = M:42; F:27	DMFT Gingival index	• MFS revealed a considerable number of enamel abnormalities, most of which were local hypoplastic spots, which may have been caused by local trauma or infection. MFS patients were more likely to have irregular pulp shape, root deformities, and pulp inclusions, especially when all three occurred together. Gingivitis was substantially worse in the MFS group than in the control group.

Mean (SD); N/A—not available.

**Table 2:** Methodological quality assessment of the studies by Newcastle–Ottawa Quality Assessment Scale (NOS).

References	Selection				Comparability		Exposure		
	1	2	3	4	5	6	7	8	9
Rahman et al., 2020 [17]	*	*	*	*	*	*	*	*	-
Laganà et al., 2019 [18]	*	*	*	*	*	*	*	*	-
Venza et al., 2019 [19]	*	*	*	*	*	*	*	*	-
Hanisch et al., 2018 [20]	*	*	*	*	*	*		*	
Dolci et al., 2016 [21]	*	*	*	-	*	*	-	*	-
Suzuki et al., 2015 [22]	*	*	*	-	*	*	*	*	-
Suzuki et al., 2014 [23]	*	*	*	-	*	*	*	*	-
Staufenbiel et al., 2013 [8]	*	*	*	-	*	*	*	*	-
De Coster et al., 2004 [24]	*	*	*	-	*	*	-	*	-
De Coster et al., 2002 [9]	*	*	*	-	*	*	-	*	-

1—Adequate case definition; 2—representativeness of the cases; 3—selections of control/comparator; 4—definitions of control/comparator; 5—case; 6—control/comparator; 7—exposure of evaluation; 8—same method for case and control; 9—non-response rate. (*): Yes; (-): No.

## Data Availability

The data used to support the findings of this study are included in the article.
